# Carbon footprint of private dental clinics in Egypt: a cross-sectional study

**DOI:** 10.1186/s12903-024-05413-0

**Published:** 2025-01-17

**Authors:** Amira H Elwan, Maha El Tantawi, Ahmed Mahmoud Fouda

**Affiliations:** 1 Department of Pediatric Dentistry and Dental Public Health, Faculty of Dentistry, Alexandria University, Alexandria, Egypt; 2 Afrone Network, Faculty of Dentistry, Alexandria University, Alexandria, Egypt; 3 Department of Fixed Prosthodontics, Faculty of Dentistry, Suez Canal University, Ismailia, Egypt

**Keywords:** Carbon footprint, Greenhouse gases, Dental clinics, Patient travel, Waste, Energy consumption, Procurement, Egypt, Africa

## Abstract

**Background:**

Climate change is a global challenge, caused by increasing greenhouse gas (GHG) emissions. Dental clinical practice contributes to these emissions through patient and staff travel, waste, energy and water consumption and procurement. Carbon footprinting quantifies GHG emissions. This study assessed the Carbon Footprint (CFP) of private dental clinics in Egypt.

**Materials and methods:**

Data were collected from private dental clinics in Alexandria and Elbeheira, in Northwestern Egypt from July to August 2024 through interview questionnaires. A CFP calculator was used to estimate carbon emissions from patient and staff travel, waste, energy and water consumption, and procurement. To determine the average CFP per clinic and per patient visit, the CFP of all clinics was averaged, both with and without considering the depreciation of dental equipment.

**Results:**

Data from 27 dental clinics were collected. The average CFP of an Egyptian private dental clinic, which, per year, received 3,322 patient visits, and where 5 personnel worked 279 days was 14,426.8 kg CO_2_e, or 4.3 kg CO_2_e per patient visit. The largest contributor to the CFP was patient travel (45.6%), followed by staff travel (19.6%), energy consumption (18%), procurement (12.4%), waste (4.2%), and water consumption (0.3%). After considering the yearly depreciation of dental equipment, the CFP per clinic in a year increased by 12.2%.

**Conclusion:**

Private dental clinics in Egypt produce substantial carbon emissions. Patient travel was the major contributor to the CFP. While there was a high CFP of electricity consumption, the CFP of gas was zero. The high CFP of waste was likely due to improper segregation and the lack of recycling. Country-specific CFP calculators are needed to accurately measure the carbon emissions of dental clinics in various settings. Preventing oral diseases, raising public awareness to sustainable practices, promoting walking and cycling, improving public transportation, implementing waste recycling, shifting to renewable sources of energy, and local manufacturing of dental products are important to reduce carbon emissions in dental clinics.

## Introduction

Human activities, such as industry and agriculture, burn fossil fuels that release carbon dioxide, which is a potent greenhouse gas (GHG) [1]. GHGs absorb and trap heat, causing global warming [2], which is the global increase in combined sea and air surface temperatures over a 30-year period [3]. Global warming is key in climate change caused by human activities leading to changes in the global atmosphere beyond the natural variation in climate [4]. Climate change leads to rising sea levels, extreme weather events and disruptions to the ecosystem [5]. Limiting global warming requires environmentally sustainable practices and continuous monitoring of GHG emissions [6, 7].

Dental clinics can be a source of GHG emissions [8] through (1) directly released emissions from sources controlled by the organization, such as combustion of fuel in vehicles owned by dental care facilities while transporting dental products, natural gas combustion for heating, and leak of nitrous oxide used in sedation [9], (2) indirect emissions from generating energy by electricity and (3) other indirect emissions, including staff and patient travel, waste disposal, water consumption, and equipment production and distribution [10].

Life Cycle Analysis (LCA) is used to assess the environmental impact of a process, including resource depletion, pollution, and carbon emissions [11]. Carbon footprinting is a subset of LCA that focuses on quantifying the direct and indirect GHG emissions, converting GHGs into a common unit called carbon dioxide equivalents (CO_2_e) [12]. Carbon footprinting can be done using Process-based Life Cycle Analysis (PB-LCA), Environmental Input-Output Life Cycle Analysis (EIO-LCA), and a hybrid method [12]. PB-LCA estimates GHG emissions by mapping the supply chain pathways and multiplying them by emission factors derived from scientific studies [13]. EIO-LCA, the spend-based approach, measures the amount of money spent on a service and multiplies it by a sector-specific emission factor [14].

The carbon footprint (CFP) of the healthcare sector worldwide is about 1.6 gigatons of CO_2_e, representing 4.4% of global GHG emissions [15], with dental care contributing to 3% of all healthcare sector’s CFP [16]. The CFP of staff and patient travel is influenced by the transportation mode. Walking or cycling have the lowest CFP. Public transportation, such as buses and trains, has less CFP per passenger-kilometer than cars [17]. The CFP of dental materials and equipment results from energy consumption for the extraction of raw materials, manufacturing, packaging, distribution and transportation [18]. Equipment constitutes a considerable portion of spending in dental clinics. The CFP of equipment is usually calculated by the EIO-LCA method, which entails that the more expensive an equipment is, such as dental units, microscopes and X ray machines, the higher its carbon emissions [14, 19]. Depreciation allocates the cost of an equipment over its expected life, thereby reflecting the consumption of its value as it is used [20]. Adding the depreciation to the CFP of procurement accounts for the yearly loss in equipment value and provides a more accurate reflection of the carbon emissions associated with the dental equipment [21]. If the CFP of procurement is calculated by the EIO-LCA, the cost of replacing dental equipment after the end of its life can inflate the CFP of dental clinics that purchased new equipment and overlook the periodic depreciation in clinics that have not yet replaced their equipment [21]. Thus, calculating the CFP associated with the annual depreciation of dental equipment should be considered although previous studies have not done so. The CFP of water includes the energy for its collection, treatment, supply services, and sewage treatment. The CFP of waste results from its transportation from the generation site to a treatment facility or incinerator, and consumes significant energy [22].

Research [23] shows that the CFP of Scotland’s National Health Service (NHS) dental services was about 160,000 tons CO_2_e (tCO_2_e) annually, representing 4% of the Scottish NHS CFP. The CFP of dental clinics, general and primary, in the NHS in England was 675,000 tCO_2_e; representing 3% of the NHS CFP in England [24]. In all cases, patient and staff travel, procurement, and energy use represented the greatest sources of CO_2_e emissions [23, 24]. The CFP of a single NHS clinic in England was calculated to be 33,438.28 kg CO_2_e [25]. Duane et al. [26] developed a calculator to monitor carbon emissions and track the sustainability of dental clinics through a hybrid model of LCA. Using the same data with the calculator, the CFP of a single NHS clinic in England was 33,119.3 kg CO_2_e, or 12.5 kg CO_2_e per patient visit.

Climate change disproportionately impacts developing countries which have limited capacity to adopt climate change mitigation strategies [27]. Africa has the lowest GHG emissions globally. However, it is the most vulnerable continent to climate change, because of its low adaptive capacity and high economic dependency on climate-related resources, such as agriculture and fishing [28]. To date, the CFP of dental clinics in African countries has not been investigated. Egypt, a lower-middle income country [29] and the third most populous country in Africa [30], is highly vulnerable to climate change [31] due to its population growth [27]. Projections indicate that Egypt will continue to experience a higher level of warming than the global average [32]. Several factors may contribute to a different CFP for dental clinics in Egypt than European countries. First, Egypt’s high temperature and humidity affect building heating, ventilation, and air conditioning (HVAC), leading to different energy consumption patterns [33]. Second, the unequal geographic distribution of dental clinics [34], dentist-to-population ratios [35], and different transportation modes [36, 37] may impact patient and staff travel CFP. Third, the lack of recycling [38], deficient incinerators, and poor waste segregation [39] could contribute to a higher waste CFP. Fourth, Egypt’s reliance on imports for most dental products from Europe involves long transportation distances [40], which could contribute to a higher CFP of procurement. Egypt has committed to reducing GHG emissions by 2030 [41]. This necessitates monitoring the GHG emissions and identification of carbon-intensive areas, including healthcare [6], and dental practice. This is particularly evident in the private sector, the fastest-growing healthcare provider in Egypt [42], where 60% of the population seek private health care which, in turn, provides 60% of the health services [43, 44]. The primary healthcare financing source is out-of-pocket (60%) with the highest spending going to private clinics (38.4%) [45].

Dental clinics in Egypt contribute to global carbon emissions and differences from clinics in High Income Countries (HICs) are assumed to exist but have not been explicitly addressed. None of the previous studies assessed the CFP of dental clinics in Low- and Middle-Income Countries (LMICs). This would help identify carbon hotspots to develop strategies for mitigating the CFP. This study aimed to estimate the CFP of private dental clinics in Northwestern Egypt.

## Materials and methods

A cross-sectional study was conducted in Elbeheira and Alexandria, in Northwestern Egypt from July to August 2024. Elbeheira has a population of 6,670,630 people, living in 9826 km² [46], whereas Alexandria, the second largest city in Egypt and its main port, has a population size of 5,441,385 people living in 2300 km² [47]. Both governorates represent 10.38% of the total population [48]. Alexandria has the highest concentration of dentists in Egypt, while Elbeheira has one of the lowest concentrations [35], yet no reliable data is available about the number of private clinics in the two governorates. Ethical approval was obtained from the Research Ethics Committee, Faculty of Dentistry, Alexandria University, Egypt (# 0941-07/2024). Informed consent was obtained from the clinic owners, dentists, and auxiliaries. Participants were informed that their data was kept anonymous and was only viewed by the study team. Data were collected from private clinics in different areas in Elbeheira and Alexandria, which included urban and rural areas. A clinic was considered eligible if it was a private, standalone clinic with at least one full-time operating dentist and a nurse. Public clinics and hospitals were excluded. Clinics within healthcare facilities were excluded because it was difficult to assess the independent water and energy consumption [23].

Interview questionnaires were used to collect year-round data on the clinics’ workflow from July 2023 to July 2024 [8]. CFP was calculated by the Duane et al. CFP calculator for dental practices [26]. Data included the average number of workdays in a year, number of full-time staff and patient visits per year, staff travel, patient travel, waste, energy and water consumption, and procurement. The yearly depreciation was calculated as per the following equation: (Dental equipment cost – salvage life) / useful life [49]. The useful life was assumed to be 5 years. The sources of data are shown in Table 1. The data of all clinics were averaged to obtain the average for a clinic, then multiplied by conversion factors to obtain the CFP in a year per clinic and the average CFP per patient visit, both with and without the depreciation cost. PB-LCA was used for all calculations of the CFP, except for procurement which was calculated by the EIO-LCA.

**Table 1:** The source of information for items used in the CFP calculator

Item	Source of information
How many days is the clinic open in an average year?	The clinic owner was asked about the average number of days the clinic was open per week and the number of days the clinic was closed during holidays.
How many full-time staff is there in the clinic?	The number of dentists, nurses, receptionists, accountants, administrative personnels, cleaning and sterilization personnel, and human resources personnel were collected.
How many patient visits for the clinic are there every year?	The clinic’s records
How far do staff travel by car to or for work in an average week? (miles)	The staff were asked about their residence location, which were cross-checked with the records. The shortest distance from their residence to the clinic, as determined by the Global Positioning System (GPS), was used.
How far do patients travel to or for the clinic by any of the following methods (petrol/diesel car, electric car, bus, train, motorbike, bike/walk) in an average week? (miles)	Thirty patients were asked about their residence locations, which were cross-checked with the records. The shortest distance from their residence to the clinic, as calculated by the GPS, was used to determine patient travel distance for each transportation method. The total distance travelled by patients was then summed for each method. The Tuk-Tuk (auto-rickshaw) is a three-wheeled transportation vehicle that is popular in less urbanized areas. It has the same engine design as the motorbike and emits equal carbon amounts [50]. The distance travelled by the Tuk-Tuk was calculated among the distance travelled by the motorbike. The tram, a popular electricity-powered mass transportation method in Alexandria, emits more carbon per passenger-kilometer than electric cars, equivalent to a diesel train [17]. The distance travelled by the tram was included in the calculations for train travel.
What is the number of waste bags that the clinic discards per week: Plastic waste for recycling, cardboard waste for recycling, infectious waste for incineration, and domestic waste for disposal (each bag weighing 6.72 kg)	The cleaning personnel were asked to count and weigh the waste bags discarded during an average week. The number of waste bags was multiplied by the average weight of the bags and then divided by 6.72 kg, which is the average weight of a filled waste bag according to the calculator [26].
How many kilowatts hour (kWh) of standard electricity, green electricity, solar power on the clinic’s roof or gas did the clinic consume last year?	Energy consumption in last year’s bills was summed. If any monthly bill was missing, the average consumption was used as a substitute for that month.
What was the water consumption last year in cubic meters (m^3^)?	Water consumption in last year’s bills was summed. If any monthly bill was missing, the average consumption was used as a substitute for that month.
How much money, in Great Britain Pound (GBP), was spent on equipment and materials during the last year? (not including rent or interest)	Last year’s invoices for dental materials, equipment, administrative costs and dental laboratory services were summed. The depreciation cost of equipment (dental units, apex locators, endodontic motors, X-ray machines, amalgamators, autoclaves, handpieces, light curing lamps, dental lasers, dental implant equipment, and digital scanners) was calculated and added to the CFP of procurement in a separate calculation. Procurement costs were converted to GBP by dividing the clinic’s expenses by the average Egyptian Pound value during 2023–2024.

## Results

Twenty-seven clinics (12 in Elbeheira and 15 in Alexandria) consented to participate in the study out of 30 invited (90%). Table 2 shows that the average number of workdays in the clinics was around 279 days per year. The average full-time staff number was 5 persons. The average number of patient visits was 3,322 per year.

In an average week, the distance the staff travelled to or for work was 115 miles, accounting for 2,833.2 kg CO_2_e per year. The average total distance travelled by patients was around 199 miles per week including 79.9 by petrol/diesel car, 92.7 by bus, 8 by train or tram, 10.2 by motorbike or Tuk-Tuk, and 8 miles on foot or by bike. Patient travel produced 6,577.7 kg CO_2_e per year, with petrol/diesel cars being the highest source of carbon emissions (4,688.5 kg CO_2_e), followed by buses (1,539.4 kg CO_2_e). An average of 1.5 infectious waste bags were collected for incineration and 1.3 domestic waste bags for disposal per week. There were no waste bags for recycling. The waste CFP was 600.4 kg CO_2_e per year.

The average electricity consumption was 9,602 kWh, accounting for 2,592.5 CO_2_e per year. No green electricity, solar power or gas were used. On average, around 110 m^3^ water was used, accounting for 37 kg CO_2_e per year. The average procurement was around 13,585 GBP, accounting for 1,785.9 kg CO_2_e per year. After considering depreciation, the average procurement was around 26,985 GBP, accounting for 3,547.7 kg CO_2_e.

**Fig. 1:**
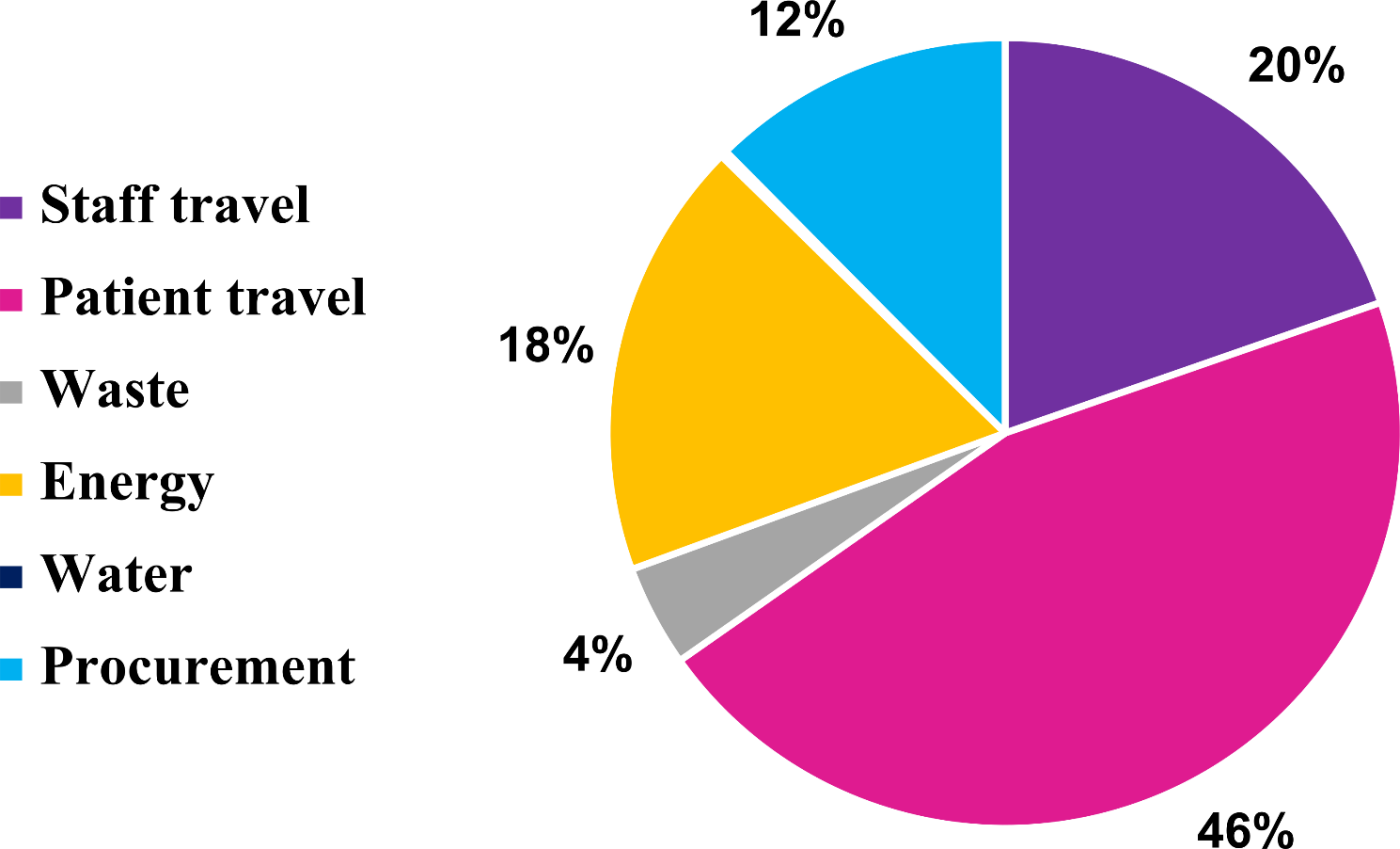
Distribution of the carbon emissions of private dental clinics in Egypt

The average total CFP of a private dental clinic in a year was 14,426.8 kg CO_2_e, 4.3 kg CO_2_e per patient visit. This rose to 16,188.5 Kg CO_2_e, 4.9 kg CO_2_e per patient visit, after adding the cost of depreciation. Depreciation increased the CFP by 12.2% **(**Table 2**)**. Patient travel represented 45.6% of the private dental clinics CFP, followed by staff travel (19.6%), energy consumption (18%), procurement (12.4%), waste (4.2%), and water consumption (0.3%) **(**Fig. 1**)**.

**Table 2:** The average CFP of private dental clinics in Egypt per year (*N* = 27)

Clinic information		Average	Conversion factors	CFP per year (Kg CO_2_e)
Number of workdays per year		278.9	-	-
Number of full-time staff		5.1	-	-
Number of patients per year		3,322.1	-	-
Staff travel in a week by car (miles)		115	0.53	2,833.2
Patient travel in a week (miles)	Petrol/diesel car	78.9	0.53	4,688.5
	Electric car	0.00	0.18	0.0
	Bus	92.7	0.15	1,539.4
	Train	8	0.19	168.8
	Motorbike	10.2	0.16	180.9
	Bike/walk	8	0.0	0.0
	Total	198.8	-	6,577.7
Number of waste bags in an average week	Plastic waste for recycling	0.0	0.0	0.0
	Cardboard for recycling	0.0	0.0	0.0
	Infectious waste for incineration	1.5	7.59	532.14
	Domestic waste for disposal	1.3	1.16	68.28
	Total	2.8	-	600.4
Energy consumption in a year (kWh)	Standard electricity	9,602	0.27	2,592.5
	Green electricity	0.0	0.01	0.0
	Solar power	0.0	0.04	0.0
	Gas	0.0	0.21	0.0
	Total	9,602	-	2,592.5
Water consumption in a year (m^3^)		109.6	0.34	37
Procurement in a year (GBP)		13,584.7	0.13	1,785.9
Procurement with depreciation in a year (GBP)		26,985.2	0.13	3,547.7
Total CFP in a year without depreciation				14,426.8
CFP in a year without depreciation per patient visit				4.3
Total CFP in a year with depreciation				16,188.5
CFP with depreciation in a year per patient visit				4.9

*CFP* Carbon footprint, *CO2e* Carbon dioxide equivalent, *kWh* kilowatt hour, *m*^*3*^ cubic meter, *GBP* Great Britain Pound

## Discussion

In private dental clinics in Northwestern Egypt, the major contributors to the CFP were patient travel, staff travel and energy consumption, whereas procurement, waste, and water had a smaller impact. The overall yearly CFP of Egyptian private dental clinics was less than half that in England (33,119 kg CO_2_e) [26]. Similarly, the CFP per patient visit in Egypt was less than that in England (12.5 kg CO_2_e) [26] and Scotland (17.7 kg CO_2_e) [23].

In this study, the number of patients’ visits was higher than in England [26], probably due to the difference in population size between the study areas with 107 million Egyptians [51] compared to 53 million in England [52]. Patients in Egypt also travelled shorter distances than in England [26], which may be due to the closeness of dental clinics in major cities like those in the study [35] and patients visiting dentists near their homes to reduce transportation costs [53]. Unlike England, Egyptian patients used buses more than private cars, possibly due to the lower rate of car ownership in Egypt than England (< 10% [54] versus 78% [55] respectively). The distances travelled by motorbikes and cycling/walking were like those in England. Electric cars were not used and are not common in Egypt [56]. Public transportation vehicles in Egypt may be older and less efficient than in England, so we may be underestimating the CFP of patient travel in Egypt [37]. It is important to invest in improving roads and public transportation for optimal fuel efficiency and to encourage energy-efficient mass transportation, such as trams and electric buses. Teledentistry should also be considered as an alternative to face-to-face appointments whenever possible [57]e.g. using videoconferencing for remote consultation, diagnosis and treatment planning, mHealth for oral health promotion, and digital imaging for screening of dental caries [58].

The private dental clinics in Egypt, not bound to a set work schedule, had a higher average number of workdays than the state-run clinics of the NHS in England. Also, the average number of full-time staff in Egyptian private clinics was greater than in NHS England, possibly due to the low salaries in Egypt [59], allowing clinics to hire more staff. More workdays and greater staff number may contribute to higher CFP of staff travel. However, the yearly CFP of staff travel was lower than in England [26] and Scotland [23]. This could be attributed to the tendency of Egyptian workers to work closer to their homes [60]. The CFP calculator assumed that staff travel was by car, although many staff members in the present study used public transport or walked. Thus, our estimates, though lower than the British estimates, may be an overestimation of the CFP of staff travel in Egypt.

The CFP of yearly energy consumption was lower than in England [26] and Scotland [23]. While standard electricity use was higher in the Egyptian than British clinics, the absence of gas use for energy contributed to a lower overall CFP. Neither green electricity nor solar panels were utilized in Egyptian clinics. Egyptian clinics relied on air conditioning during summer due to high temperatures and humidity, while heating was rarely needed in winter. Conversely, the British clinics used heating for most of the year [61]. Egypt heavily relies on the nonrenewable energy of fossil fuels [62]. Transitioning to renewable energy is crucial to mitigate carbon emissions [10]. Well-designed buildings of healthcare facilities would significantly reduce air conditioning use with the associated GHG emissions [63, 64]. This requires financial resources, access to recent technology, proper power grids, and efficient energy distribution networks [65].

The CFP of yearly water use was more than double that of England [26], possibly due to the higher number of patients in Egypt. More patients consume more water from dental units, handpieces, ultrasonic scalers, infection control measures, and domestic use [22]. Also, Egypt’s aging water pipelines may cause a water leak, resulting in a higher water CFP [66].

The CFP of yearly procurement was considerably lower than in England [26]. Duane et al. [25] calculated the CFP of procurement by a spend-based approach and used a conversion factor based on the total carbon emissions of the NHS expenses, adjusted for inflation. This generic conversion factor may not accurately reflect the CFP of procurement in other countries, such as Egypt, because of different inflation rates, oral health care expenditure, and supply chains. The inflation rate, at present, is 2.2% in England and 25.7% in Egypt [67]. The UK allocates more annual funding to oral health than Egypt (3 billion GBP versus 0.257 ≈ 0.3 billion GBP) [68, 69]. Also, Egypt imports most dental products from other countries, resulting in longer transportation distances [40, 70]. These differences in the CFP components for procurement between countries emphasize the need to develop customized calculators to assess the CFP of dental clinics in different settings.

Estimating the CFP of procurement based solely on product prices may inflate the CFP of newly purchased units and devices. The replacement rate of dental equipment is expected to be higher in the UK due to greater funding [68, 71]. Calculating the CFP taking into consideration the depreciation of existing equipment resulted in a notable increase in the CFP of procurement. This underscores the importance of investing in equipment with extended service life, as such equipment reduces the need for frequent replacements, saving costs associated with purchasing, transporting, and installing new equipment. This ultimately contributes to more sustainable value chains and improved environmental efficiency [21]. Additionally, each country should manufacture their own dental products, as this will shorten the supply chain and eliminate the carbon emissions associated with cross-country transport.

The CFP of yearly waste was higher than in England [26], possibly due to the limited recycling and inadequate segregation of waste [39, 72]. Reusing dental instruments after thorough cleaning, disinfection, and sterilization helps reduce waste CFP [73]. Also, recycling dental materials reduces the demand for raw materials and minimizes waste disposal and incineration [74]. In addition, there is a need to rethink the entire supply chain [75] to move away from the linear economy model [76], characterized by the consumption and disposal of products, leading to resources loss, high procurement rates, and end-of-life waste [77]. Instead, circular economy prioritizes reducing, reusing and recycling resources throughout production, distribution, and consumption [78], thus, decreasing the CFP of waste and procurement along the supply chain [10].

The study has some limitations. First, the Duane CFP calculator was designed with assumptions fitting the UK setting and does not accurately reflect the carbon emissions in Egyptian dental clinics. Second, the CFP of procurement and waste may not have been precisely measured due to the complexities and uncertainties of dental products’ supply chains and waste weighing [25]. Third, as the CFP estimation is typically limited to a specific timeframe (one year) [8], it did not account for carbon emissions from the construction or maintenance of dental clinics, as none were recently built or maintained. Nevertheless, the study has several strengths. First, the carbon footprinting of patient travel considered country-specific transportation modes, such as the Tuk-Tuk, which had lower carbon emissions than some vehicles. Second, the calculation of dental equipment depreciation identified a previously overlooked source of GHG emissions that accumulates over the equipment lifespan. Third, the present study estimates the CFP of dental clinics outside Europe and the Global North for the first time, thus providing a perspective into the challenges of sustainable dental practice in LMICs.

As demand for oral healthcare increases, the associated carbon emissions will escalate, if oral diseases are not sustainably managed [10]. Preventing oral diseases remains the most effective strategy for reducing carbon emissions, as it minimizes follow-up visits and associated waste [18]. Preventive programs, such as oral health education, community water fluoridation, and school dental sealants programs are strongly recommended in this respect with incentivization of prevention-based oral healthcare, integration of dental care to primary health care, and providing dental public health specialty training programs [79, 80]. Oral healthcare providers should be educated about carbon reduction interventions and encouraged to regularly report on their clinics’ carbon emissions. Environmental protection agencies should adopt environmental standards, ensure that dental clinics implement GHG mitigation measures, and take legal actions against those who do not conform. The academic community should assess context-specific solutions to manage carbon emissions from dental clinics [81].

Sustainable Development Goals recognize that action in one area will affect outcomes in others [82]. Reducing carbon emissions resulting from dental clinics can mitigate climate change and the associated health consequences [10]. It is important to formulate policy and guidelines that integrate sustainable practice to healthcare delivery and consider both planetary and community dental health [83]. LMICs can benefit from some carbon mitigation strategies such as, encouraging dental staff to walk or cycle to work [84], using energy-efficient appliances (LED lights, fluorescent lighting, and energy-saving air conditioning), purchasing goods with minimal packaging, minimizing the use of paper for patient records, reducing the reliance on single-use plastic products (SUPs) [85], and fostering small and medium-sized enterprises (SMEs) which develop affordable recycling programs [86] and waste exchange platforms [87]. Barriers to sustainability implementation in LMICs should also be carefully considered. These include the public’s limited knowledge, attitudes, and practices regarding sustainability [88], unaffordability of clean energy, high transition costs [89], and the lack of comprehensive sustainability policies and guidelines [90].

Future studies are needed to develop country-specific CFP calculators, establish methodologies to address inherent inaccuracies in waste weighing, travel distances, and procurement, evaluate the effectiveness of interventions aimed at reducing carbon emissions, and analyze the life cycle of various dental procedures to quantify their environmental impact.

## Conclusion

Private dental clinics in Egypt produce less carbon emissions than dental clinics in high income countries. Patient travel was the primary contributor to these emissions. One of the main differences in the CFP profile of dental clinics in Egypt was that the CFP of gas was zero as no heating was used in winter. However, there was a high electricity CFP. Another difference was the high CFP of waste because there was no recycling or proper segregation. It is important to develop context or country-specific CFP calculators to measure the CFP of dental clinics in various settings. Carbon emissions of dental clinics in LMICs can be reduced by dental practice-specific measures, such as preventing oral diseases, local manufacturing of dental products, and minimizing the use of SUPs. Additionally, national-level, multi-sectoral measures can play a vital role, including raising public awareness, improving public transportation, shifting to renewable energy, adopting a circular economy, promoting walking and cycling, using energy-efficient appliances, and fostering small waste recycling programs and waste exchange platforms.

## Data Availability

The dataset used and/or analyzed during the present study is available from the corresponding author on reasonable request.

## Abbreviations

GHG: Greenhouse Gas
LCA: Life cycle Analysis
CFP: Carbon Footprint
CO_2_e: Carbon Dioxide Equivalent
PB-LCA: Process-based Life Cycle Analysis
EIO-LCA: Environmental Input Output Life Cycle Analysis
NHS: National Health Service
PHE: Public Health England
Kg: Kilogram
HVAC: Heating, ventilation, air conditioning
GPS: Global Positioning System
kWh: Kilo watthour
m^3^: Cubic meter
GBP: Great Britain Pound
LMICs: Low- and middle-income countries
SUPs: Single Use Plastic Products
SMEs: Small and Medium Size Enterprises
